# The change of working alliance and the association to treatment outcome in an internet-based therapy after pregnancy loss

**DOI:** 10.1186/s40359-024-01751-4

**Published:** 2024-05-07

**Authors:** Martin Kramuschke, Jana Reinhardt, Ruth Dölemeyer, Julia Kaiser, Anette Kersting

**Affiliations:** Department of Psychosomatic Medicine and Psychotherapy, University of Leipzig, Semmelweisstraße 10, 04103 Leipzig, Germany

**Keywords:** Working alliance, Therapeutic relationship, Internet-based therapy, Cognitive behavioral therapy, Pregnancy loss, Perinatal loss, Grief, Prolonged grief, Traumatic stress

## Abstract

**Background:**

Working alliance is a prominent non-specific factor for treatment outcomes in face-to-face and internet-based interventions. The association between working alliance and therapy outcome appears to be time- and disorder-specific, but less is known about the change of working alliance during the intervention and the impact of working alliance in grief-specific interventions. The present study examines the association between the change of working alliance and treatment outcomes in an internet-based intervention for parents who experienced pregnancy loss.

**Methods:**

228 participants received a grief intervention based on cognitive behavioral therapy with asynchronous text-based therapist feedback. Prolonged grief and related symptoms of traumatic stress, depression, anxiety, and general psychopathology were assessed with validated instruments before and after the intervention. The change of working alliance was assessed using the short version of the Working Alliance Inventory at mid-treatment (session 4) and the end of the treatment (session 10).

**Results:**

Data for *N* = 146 persons was analyzed. Working alliance in total and all subscales increased significantly from sessions 4 to 10. This change in working alliance correlated significantly with a reduction in prolonged grief. Changes in subscales of working alliance also correlated with symptoms of depression and general psychopathology. Regression analysis showed that a change in working alliance predicted a reduction in prolonged grief but did not predict improvements in other grief-related symptoms.

**Conclusion:**

The results examine the change of working alliance during an internet-based intervention and the association with treatment outcome. A small impact of change in working alliance on treatment outcome of prolonged grief was confirmed, but not on related symptoms. Further research is needed to assess moderators of the alliance-outcome association to improve internet-based interventions.

**Trial registration:**

Not applicable.

**Supplementary Information:**

The online version contains supplementary material available at 10.1186/s40359-024-01751-4.

## Background

The experience of perinatal loss can be traumatic, often resulting in intensive grief and post-traumatic stress symptoms [1]. Despite a growing awareness of the emotional and psychiatric impact of perinatal loss, few affected parents receive therapeutic intervention, and manualized interventions tailored for this group have rarely been evaluated [1–3].

Internet-based interventions offer a promising approach, enhancing healthcare services by offering an additional, supplemental, and cost-effective path to delivering those services. Interventions conducted via the internet provide more anonymity, privacy, and flexibility compared to face-to-face interventions. This makes them particularly appealing for clients or patients who fear stigmatization or are difficult to reach [4–6].

In order to comprehend and improve online treatment outcomes, it is crucial to identify common and specific factors that influence those outcomes [7]. The therapeutic relationship is considered an important factor impacting the success of therapy in face-to-face studies [8–10]. It is represented in part by the construct of therapeutic alliance [11, 12], which is often conceptualized as *working alliance* [13–16]. The term working alliance (WA) encompasses the quality and strength of the relationship between client and therapist, incorporating three interlocking components: *task*, *goal*, and *bond* [14–16]. The *bond* dimension describes the emotional aspects of the relationship between therapist and patient (i.e. level of trust, respect, and caring). The *task* dimension refers to the agreement on tasks in the process, whereas the *goal* dimension refers to an accord on implicit or explicit aims of the treatment [14, 15, 17].

The association between WA and treatment outcome in internet-based interventions has undergone extensive examination [13, 18] and shows a small but robust effect size, quantitatively similar (*r* = .202–0.275) [19–21, 13] to face-to-face interventions (*r* = .278) [9, 13, 22]. Meta-analytical data indicates minor variations in the alliance-outcome association. These variations might occur due to factors related to the intervention, characteristics of the sample involved, or the methods employed [19, 21].

Kaiser et al. [19] identified point of data collection (early-, mid-, post-treatment) of WA as the only significant moderator affecting the association between WA and treatment outcome in internet-based interventions. In general, WA showed greater robustness and was correlated more often with treatment outcomes when measured at the end of the internet-based treatment [19]. This observation suggests that WA may develop progressively throughout treatment and grows linearly. However, this assumption was not directly tested in internet-based studies.

To the best of our knowledge, all studies included in the aforementioned meta-analytical studies [19, 21] only examined the correlation of WA with outcome based on one point of data collection, e.g., mid-treatment(e.g., [26, 27]) or post-treatment (e.g., [28, 29]), or correlated an average WA score (composite score) with outcome measures [29, 30]. Findings were mixed, revealing either an association [29, 30] or no association between WA and improvement in treatment outcomes [31].

We found no study calculating the change in WA and its correlation with treatment outcome. One possible explanation might be that WA scores did not change during the intervention (e.g., 33). In other studies, the observed variations in WA were not treated as a separate predictor for treatment outcome (e.g., 31). Assessing the change in WA throughout treatment enhances our understanding of the association between WA and treatment outcomes in internet-based interventions. In contrast to other studies, the present study focuses on the change in WA during an internet-based intervention.

The treated diagnoses and corresponding symptoms may influence to what extent WA is associated with treatment outcomes in internet-based interventions similar to face-to-face interventions [13, 33]. Literature remains scarce about the evaluation of WA in grief-specific internet-based interventions, despite data from face-to-face therapies highlighting the important role of WA in the treatment of prolonged grief [34, 35]. To the best of our knowledge, just one further study evaluated the association of WA and treatment outcome in an internet-based treatment program using internet-delivered writing assignments [36]. This study used therapist-rated instead of patient-rated WA. Although patient and therapist ratings seem to be associated similarly with treatment outcomes [13], the study did not find a significant association between WA and grief [36]. We aim to investigate the association of patient-rated WA and grief-specific outcomes to enhance existing findings.

Grief symptoms exhibit both similarities and differences with related syndromes, like posttraumatic stress disorder (PTSD), depression, and anxiety [35, 37]. Changes in WA might also correlate with these syndromes. Internet-based grief interventions were found effective in reducing grief and PTSD symptoms, with moderate to large effect sizes, yet appeared less effective in reducing depression symptoms, with small effect sizes [38, 39]. Network analysis revealed higher levels of symptom overlap between prolonged grief and PTSD compared to depression [40]. These findings indicate that WA is less associated with depression and anxiety than PTSD.

In our study, we focus on grief symptoms that occurred after pregnancy loss. Grief reactions after prenatal loss may differ slightly from other forms of bereavement: Cognitive processing has been found to primarily involve guilt-related content [41–43], and a reduced occurrence of mourning rituals has been found [44]. However, the intensity of grief after perinatal loss might be comparable to grief following other forms of loss [1].

In addition to point of data collection and the treated symptoms involved, research also shows that task, goal, and bond related-aspects of WA may demonstrate different associations to treatment outcome. Bond related aspects might be less important than goal- and task-oriented aspects, due to remote contact and reduced interaction in internet-based interventions [18, 21]. Studies indicated that task-related aspects of WA correlated significantly higher with treatment outcomes than bond-related aspects [21]. Other studies report high bond-related ratings early in the program [18]. Our study aims to clarify the question of how various aspects of the WA are associated with treatment outcomes [45].

### Aims and hypothesis

This study seeks to analyze the association of changes in WA with symptom outcomes of an internet-based intervention for grief reactions after prenatal loss. Our first hypothesis predicts a significant increase in WA during the treatment [1]. Additionally we hypothesized: An increase in WA during the treatment correlates with a reduction in grief symptoms as the primary outcome [2], and the increase in WA is predictive of grief reduction [3]. Task- and goal-related aspects of WA are more important for the reduction in grief symptoms than bond-related aspects [4]. We expected that an increase in WA is also correlated with secondary outcomes like a reduction in traumatic reactions [5] and an improvement of global mental health [6], but less with a reduction in depressive [7] or anxious reactions [8, 38, 39].

## Methods

The present study focuses on the impact of changes in WA in an internet-based cognitive behavioral intervention on grief symptoms after pregnancy loss. It is a secondary analysis of data presented in Kersting et al. [46]. Primary outcome analyses showed that an internet-based cognitive behavioral intervention significantly reduced symptoms of prolonged grief, traumatic stress, and overall psychopathology [46, 47].

### Participants

The internet-based cognitive behavioral intervention was offered to parents who had lost a child during pregnancy. Information about the study was distributed with the help of medical practitioners and midwives, who informed potential participants. Study information was also published through radio, TV, internet, and newspaper announcements.

Inclusion criteria for participating were [1] loss of a child during pregnancy through miscarriage, termination of pregnancy due to medical indication, or stillbirth at any time in the past [2], living in a European German-speaking country [3], being able to read and write the German language fluently [4], having access to the internet, and [5] signing informed consent. Exclusion criteria were [1] age < 18 years [2], having a current pregnancy [3], being in psychotherapy, and [4] suffering from serious mental health problems, like severely depressed mood, suicidal tendencies, dissociative tendencies, psychotic symptoms, or substance abuse or dependence. These criteria were assessed with validated instruments via an online screening procedure. Participants who did not meet the criteria were offered guidance towards suitable treatment options and assistance in finding treatment if requested. For a more detailed description of the recruitment process, see Kersting et al. [46, p. 373].

After the recruitment process, participants were randomly assigned to a treatment group (TG; *N* = 115) or a waiting control group (WCG; *N* = 113). Participants in the waiting group received the treatment five weeks later.

### Treatment

The intervention is based on the *Interapy program* [48], which has been evaluated several times [49, 50]. It is an asynchronous text-based and therapist-assisted cognitive behavioral therapy program for PTSD, consisting of 10 writing assignments based on the written disclosure procedure [51]. The internet-based intervention was later adapted for prolonged grief [52] and could achieve a reduction of prolonged grief within a medium effect size (Cohen’s *d* = 0.52).

The intervention in this study consisted of 10 writing tasks, divided into three sections: [1] self-confrontation (4 writing assignments) [2], cognitive reappraisal (4 writing assignments), and [3] social sharing (2 writing assignments). The first Sect [1]. comprised repeated detailed (cognitive, emotional, and sensory) self-confrontation with the loss. In the second section of cognitive reappraisal [2], participants repeatedly wrote a letter to a hypothetical friend who experienced a similar loss. They were encouraged to change their perspective and reflect on assumptions, dysfunctional automatic thinking or behavior, and feelings of guilt. In the last Sect [3]., participants wrote a letter to a significant other or themselves, outlined the most difficult memory of the pregnancy loss, reflected upon the therapeutic process, and explained their coping with the loss in the future. Communication between therapists and patients was asynchronous and exclusively text-based, consisting of personal feedback by e-mail after every second writing task from sessions 1 to 8 and every writing task after the 9th and 10th task. Participants were instructed to spend approximately 45 min per writing task. Participants were encouraged to write or call the therapist only in case of crisis and distress. Contacts beyond the writing assignments occurred infrequently. The exact number of contacts and the therapist’s characteristics were not coded. The majority of therapists participated in a training program to become licensed psychotherapists and were supervised regularly by the licensed psychotherapists working in this project. On average they treated 20.7 participants (*SD* = 13.2; see Table A).

### Measures

The outcome measures were prolonged grief, traumatic stress, depression, anxiety, and global mental health. Symptoms were assessed using self-report questionnaires at baseline, post-treatment, and follow-ups 3 and 12 months after the end of treatment. As participants completed the short form of the Working Alliance Inventory [53, 54] at mid-treatment after two weeks (after session 4) and at post-treatment (after session 10), analysis of the outcome scores in the present study includes only pre-treatment and post-treatment. A detailed description and the psychometric properties of the questionnaires are mentioned below.

### Working alliance

The working alliance was measured with the short form of the Working Alliance Inventory (WAI-S) [53]. There was no validated German version of this questionnaire. The German translation was developed by Knaevelsrud and Maercker [28, 55]. 12 items are rated on a 7-point Likert scale (1 = “never” to 7 = “always”). Higher scores indicate a better quality of the WA. A total score (ranging from 12 to 84) comprises three subscales (goal, task, and bond). The three subscales correlate highly with each other. The factor structure of the short form has not been validated [60]. Recent work used the revised form of the Working Alliance Inventory (WAI-SR) [60]. At the time of assessment of this study, the translated and validated form of the WAI-SR was not yet available. Nevertheless, the WAI-S is a reliable measure of the therapeutic alliance [61]. The internal consistency of the total scale of the WAI-S in the present study was good, ranging from Cronbach’s *α* = 0.80 at mid-treatment to Cronbach’s *α* = 0.85 at post-treatment.

### Prolonged grief

Prolonged grief was measured using the German version of the Inventory of Complicated Grief (ICG) [56]. The ICG measures emotions, cognitions, and behaviors concerning grief. It consists of 15 items rated on a five-point measurement scale (1 = “almost never” to 5 = “always”). The ICG has been shown to have good to excellent psychometric properties with a Cronbach’s α of 0.94 [56]. In the present study, Cronbach’s *α* was 0.89 at pre-test and 0.91 at post-test.

### Traumatic stress

Traumatic stress was assessed by the German version of the Impact of Event Scale-Revised (IES-R) [57]. Based on 22 items, a total score and the three subscales *intrusion*, *avoidance*, and *hyperarousal* can be calculated. The frequency of each symptom over the last seven days is assessed on a four-point Likert scale (0 = “not at all”, 1 = “sometimes”, 3 = “rarely”, 5 = “often”). The IES-R has been shown good to excellent psychometric properties with Cronbach’s *α* = 0.90 [57]. In the present study, Cronbach’s α of the total IES-R score ranged from 0.84 at pre-test to 0.90 at post-test.

### General psychopathology, depression, and anxiety

The German version of the Brief Symptom Inventory (BSI) [58] was used to measure global mental health, depression, and anxiety symptoms. The BSI consists of 53 items, assessing the experience of physical and psychological symptoms on a five-point Likert scale (0 = “not at all” to 4 = “very often”). Nine primary symptoms indices can be calculated, including depression and anxiety addressed in this study. Their sum provides a Global Severity Index (GSI) of overall mental health. In the present study, Cronbach’s *α* of the GSI score ranged from 0.94 at pre-test to 0.96 at post-test. Cronbach’s *α* for the subscale *depression* was 0.83 at pre-test and post-test. Cronbach’s *α* for the subscale *anxiety* was 0.77 pre- and respectively 0.82 at post-test.

### Sociodemographic and obstetric data

The socio-demographic data collected included age, gender, level of education based on the highest school qualification (low, medium, high), years of education, and employment status. In addition, the strength of religiosity and faith was assessed (“How strong would you describe your religiosity or faith?“). The item was rated on a 5-point Likert scale (1 = “not at all” to 5 = “very strong”). The time since the prenatal loss, the pregnancy week during which the loss occurred, and the number of previous miscarriages were recorded as obstetric data and included in the sample description.

### Statistical analysis

The original data set contained data from a treatment group and a waiting control group (see 47). Both groups received the intervention. The treatment group started the intervention right after the baseline survey. For ethical reasons, the waiting control group received the intervention five weeks later. The waiting group completed a baseline survey before the waiting period and a pre-treatment survey after the waiting period. The data before and after treatment from both groups was merged to increase the statistical power of subsequent tests. Post hoc analysis was conducted using t-tests and chi-square tests to examine significant differences between the two groups.

Dropouts and missing values were identified afterwards. A distinction was made between three types of dropouts: Nonstarters who did not start the intervention [59], dropouts who did not complete the intervention or the post-measurement, and dropouts who did not establish a complete data set due to missing values on a whole scale.

Missing values at item level were then analyzed and classified in the final analysis sample. The MCAR Little test was chosen to evaluate missing values of all scales [60]. After evaluating missing values, they were computed by the participant’s mean of the scales or subscales [61].

Means and standard deviation of the WAI-S total score, its subscales, and the primary outcome were calculated for all measurement points (pre- and post-treatment, respectively mid- and post-treatment in the case of WA). Possible differences in the mean values between the treatment group and waiting control group were checked using two-tailed t-test and chi-square test. Based on non-existent mean differences, the aforementioned merging procedure was acceptable.

Dependent two-tailed t-tests were conducted to account for within-group differences. Effect sizes for repeated measurement *d*_*Repeated Measures*_*(d*_*RM*_) were calculated [62]. Absolute change scores were calculated to account for changes in the WA throughout therapy. They were reliable [63, 64] and superior to other relative change scores [65]. Statistical analysis included the correlation of change in WA with the outcome change scores. As the predictor changes in WA and changes in the BSI were not normally distributed, the bivariate Spearman correlation coefficient, a more conservative nonparametric measure, was used. WAI-S absolute change scores (post- minus mid-treatment) were correlated with the absolute change scores in symptomatology of grief (ICG), posttraumatic stress (IES-R), depression (BSI), anxiety (BSI) as well as global mental health (BSI) (each post- minus pre-treatment scores). Effect sizes for repeated measurements were calculated for the differences in pre- and post-scores of all variables [62].

Additionally, hierarchical regression analyses were calculated for the outcome measures to estimate the influence of change in WA on treatment outcome. Only variables significantly correlating with changes in WA were included in the regression analysis. Based on outlier analyses, data points that were three standard deviations below or above the mean of each variable were eliminated [66]. We tested for autocorrelation, normal distribution of residues, and multicollinearity. Requirements for regression analysis were fulfilled.

The absolute change scores of the primary (ICG) and secondary outcomes (BSI, IES-R) were used as dependent variables, and the absolute change scores of the WA (WAI-S) were used as independent variables. The pre-scores of the corresponding outcome variables were used as independent variables to control for initial symptom levels. A single hierarchical regression model was calculated for each outcome variable and each subscale of the WAI-S. Based on theoretical reasons, multicollinearity was assumed among the subscales, which was then also present in the data set. Therefore, the subscales were analyzed in separate regression models.

All analyses were conducted using SPSS 27. The significance level was set at α = 0.05 for all statistical analysis. Taking into account the multiple testing in t-tests, correlation, and regression analysis, the Benjamini and Hochberg correction was used to adjust for the false discovery rate [67].

## Results

### Attrition

**Fig. 1:**
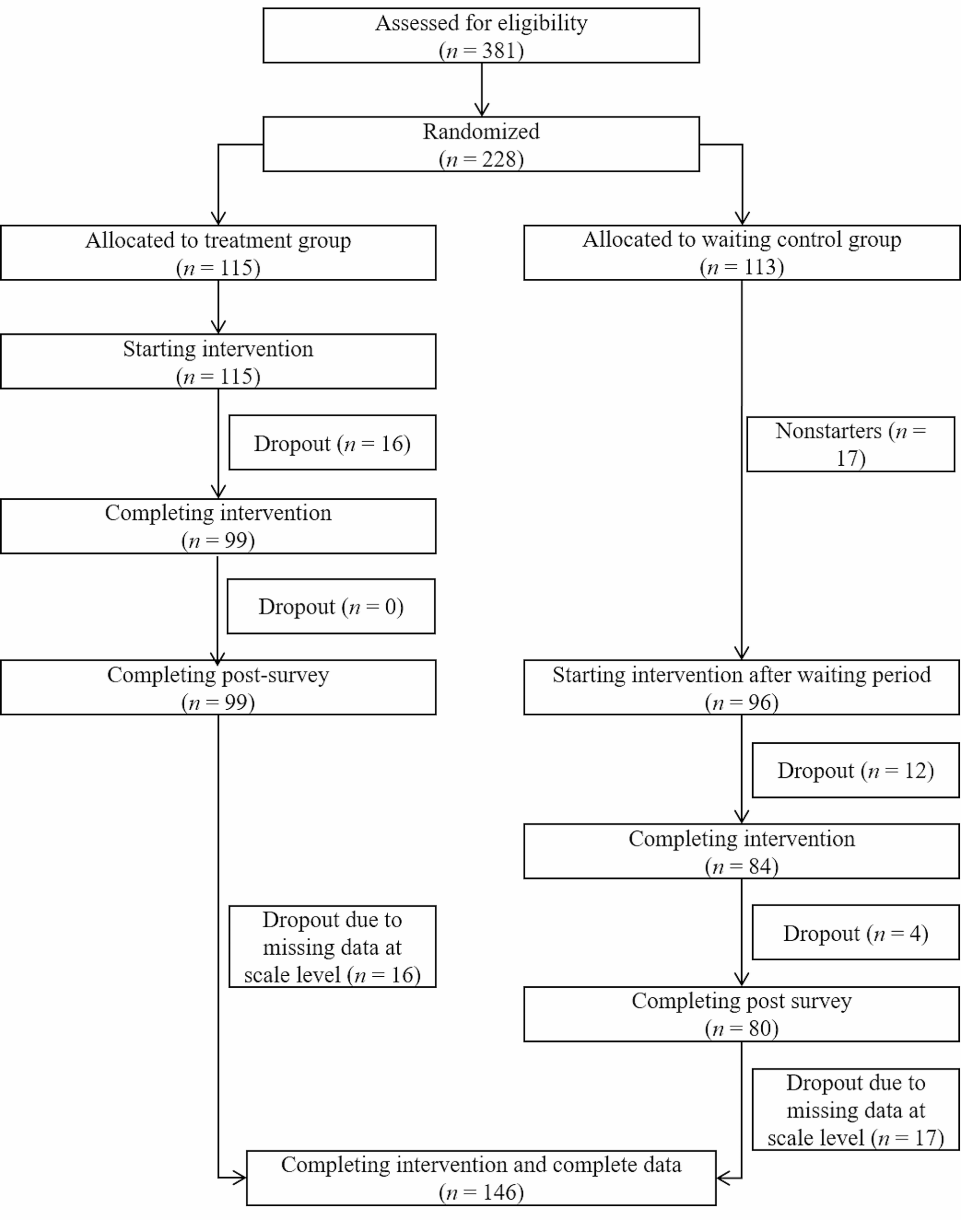
Participants’ progression flowchart

After the screening (*N* = 381), 228 participants met the inclusion criteria, gave informed consent, and were randomized into a treatment and waiting control group (see flowchart Fig. 1). Of the 228 participants who completed the baseline survey, 17 participants in the waiting control group left the program without participating in the intervention (nonstarters) [59]. 32 participants out of both groups dropped out of the intervention or did not complete the post-survey (referred to as dropouts). 32 participants did not answer at least one questionnaire before or after treatment. One more participant was assigned to this group due to missing data at scale level (15 items missing in a row).

Participants who dropped out before the intervention, respectively before the post-survey, were younger (*t*(43.82) = 2.76, *p* = .008) and less religious (*t*(53.44) = 2.10, *p* = .041) than completers. In terms of obstetric data, dropouts lost their child significantly earlier in pregnancy (*t*(163.37) = 2.14, *p* = .034). No significant differences between these dropouts and completers were found in any other baseline measures or in the mid-treatment score of working alliance (see appendix, Table B). Dropout due to missing values on an entire questionnaire or its subscales did not differ significantly from the mean values of the completers in any of the variables examined (see appendix, Table C).

### Missing values at item-level

Missing values from the analyzed sample of *N* = 146 were further evaluated. The number of missing values per scale is described in the appendix (see Table D). First, Little’s MCAR test was used to obtain an initial indication of the distribution of missing values in relation to the total scales. The missing completely at random (MCAR) assumption was rejected, as the test was significant for both the BSI total scale and the WAI-S post scale. However, the missing values of the BSI scale were not subject to any notable pattern. At item level, three items had two missing values each. The contents of the items did not provide sufficient information to make conclusions regarding missing values based on the missing at random (MAR) or missing not at random (MNAR) assumption. Missing values were therefore imputed with the person mean [61].

In the distribution of missing values in terms of the WAI-S, it is apparent that there were more missing values after the 4th writing task at mid-treatment (first point of data collection). It is possible that participants skipped items because they might have felt unable to assess the alliance at this early stage of the process. However, no noticeable pattern of missing values could be identified at the subscale level. An imputation of the missing values by the participant’s mean values was also seen to be tolerable [61].

### Participants

Participants’ ages ranged from 25 to 45, with a mean age of *M* = 34.53 (*SD* = 4.91). 91.8% (*n* = 134) were female, 8.2% (*n* = 12) were male. No differences between the treatment group (TG, *n* = 83) and the waiting control group (WCG, *n* = 63) were found for demographic as well as obstetric data, as can be seen in the outcome paper of Kersting, et al. [46]. In addition, the mean values of the two groups did not differ regarding any outcome measure before and after the intervention (see appendix, Table E). The matching of data of both groups was considered to be acceptable.

### Changes in symptoms and working alliance

Mean scores of all t-tests of the WAI-S scores, its subscales, and the effect sizes for repeated measurements *d*_*RM*_ [62] are shown in Table 1.

**Table 1:** WAI-S mean scores, results of the t-tests and effect sizes

WAI-S	*N*	*M*_*mid*_	*SD*_*mid*_	*M*_*post*_	*SD*_*post*_	*t*	*d*_*RM*_	*M*_*Δ*_	*SD*_*Δ*_
Total Score	146	5.87	0.80	6.14	0.69	-6.43***	0.52	0.26	0.49
**Subscales**									
Goal	146	6.02	0.85	6.33	0.66	-5.42***	0.41	0.31	0.68
Task	146	5.74	0.89	5.98	0.81	-4.43***	0.35	0.24	0.64
Bond	146	5.85	0.96	6.10	0.83	-4.73***	0.37	0.25	0.64

Note. WAI-S = Working Alliance Inventory short form; *M*_*mid*_ = mean score WAI-S after session 4; *M*_*post*_ = mean score WAI-S after session 10; *d*_*RM*_ = d_Repeated Measures_, M_*Δ*_ = mean score of absolute change; Significance with alpha correction: ***p < .001, **p < .01, *p < .05

The mean scores of all t-tests of the primary and secondary outcome scores can be found in Table 2. All t-tests indicated that pre- and post-scores differed significantly (*p* < .001), i.e., WAI-S scores increased and all outcome scores decreased. The average rating of WAI-S was close to the maximum of 7. Therefore, a ceiling effect is to be assumed, especially at post-treatment.

**Table 2:** Outcome mean scores, results of the t-tests and effect sizes

Outcome	*N*	M_pre_	SD_pre_	M_post_	SD_post_	t	d_RM_
ICG	146	2.47	0.61	1.94	0.50	13.33***	-1.03
IES-R_total_	146	1.86	0.69	0.91	0.64	15.99***	-1.28
IES-R_intru_	146	2.41	0.95	1.31	0.92	12.98***	-1.06
IES-R_hyper_	146	1.65	0.97	0.73	0.77	12.04***	-0.91
IES-R_avoid_	146	1.51	1.03	0.68	0.67	11.11***	-0.81
BSI_depression_	146	1.08	0.79	0.49	0.53	9.64***	-0.70
BSI_anxiety_	146	0.68	0.69	0.31	0.38	7.33***	-0.52
BSI_GSI_	146	0.80	0.53	0.39	0.36	13.33***	-0.77

*Note. M*_*pre*_ = mean score before treatment; *M*_*post*_ =mean score after treatment; ICG = Inventory of Complicated Grief; IES-R_total_ = Impact of Event Scale revised total score; IES-R_intru_ = IES-R subscale Intrusion; IES-R_hyper_ = IES-R subscale Hyperarousal; IES-R_avoid_ = IES-R subscale Avoidance; BSI = Brief Symptom Inventory; GSI = Global Severity Index; *d*_*RM*_ = d_Repeated Measures_; Significance with alpha correction: ***p < .001, **p < .01, *p < .05

### Correlations of outcome and changes in the working alliance

Spearman correlations between the change in the WA and the change in the outcomes are presented in Table 3.

**Table 3:** Spearman correlations between the change in working alliance and the change in the outcomes

Change Scores	ΔICG_total_	ΔIES_total_	ΔIES_intru_	ΔIES_hyper_	ΔIES_avoid_	ΔBSI_depression_	ΔBSI_anxiety_	ΔBSI_GSI_
ΔWAI-S_total_	0.34***	0.09	0.10	-0.01	0.18	0.24*	0.13	0.24*
ΔWAI-S_goal_	0.27**	0.08	0.08	-0.01	0.19	0.25*	0.22*	0.28**
ΔWAI-S_task_	0.24*	0.09	0.08	-0.03	0.15	0.14	0.01	0.11
ΔWAI-S_bond_	0.29**	-0.01	0.03	-0.01	0.05	0.22*	0.11	0.24*

Note. Δ = Absolute Change Scores; WAI-S = Working Alliance Inventory short form; ICG_total_ = Inventory of Complicated Grief; IES_total_ = Impact of Event Scale total score; IES_intru_ = IES subscale Intrusion; IES_hyper_ = IES subscale Hyperarousal; IES_avoid_ = IES subscale Avoidance; BSI_GSI_ = BSI Global Severity Index. Significance with alpha correction: ***p < .001, **p < .01, *p < .05.

The WAI-S change score and all change scores of the subscales correlated significantly with the ICG change score (for Spearman’s *ρ* of the subscales, see Table 3). Concerning the IES-R, there was no significant correlation between the WAI-S change score and the IES-R change scores. Concerning the BSI, significant correlations were found for the total WAI-S change score, changes in the subscale of goal, and changes in the subscale of bond with the BSI subscale *depression* and the BSI total scale of *global mental health*. Furthermore, the change score of the goal subscale of WAI-S correlated significantly with the change score of the BSI *anxiety* subscale (Spearman’s *ρ* = 0.22, *p* = .021). All correlations indicate as the WA increases, there is a corresponding reduction in symptoms.

### Prediction of treatment outcome

Hierarchical regression analyses were used to test the influence of changes in WA on the symptom level changes in case the change in WA correlated significantly with the outcome variables. The pre-treatment score was entered in a first step to control for the initial symptom level. In the second step, the WAI-S change score was entered. Results are shown in Table 4 and the appendix (see Table F).

After controlling for the initial ICG total score (adjusted *R*^*2*^ = 0.36, *F*[1,144] = 82.66, *p* < .001), the change in the WAI-S total score significantly predicted treatment outcome (adjusted *R*^*2*^ = 0.39, F[2,143] = 47.73, *p* < .001). Similarly, the change scores of the subscales *bond* (adjusted *R*^*2*^ = 0.38, *F*[2,143] = 45.93, *p* < .001) predicted treatment outcome as well. Results of the subscales can be found in Table 4.

**Table 4:** Prediction model of changes of outcome variables by changes in working alliance

Variable	Block	Predictor	*n*	*R*^2^adj.	ΔR^2^	*p*_F−Change_	F	*p*	B	SE	β	*p*
ΔICG	1	ICG_pre_	146	0.360	0.365	< 0.001	82.66	< 0.001	0.47**	0.05	0.60	0.001
	2	ICG_pre_	146	0.392	0.036	0.004	47.73	< 0.001	0.44**	0.05	0.56	0.001
		ΔWAI-S_total_	146						0.19**	0.07	0.20	0.006
ΔICG	1	ICG_pre_	146	0.360	0.365	< 0.001	82.66	< 0.001	0.47**	0.05	0.60	0.001
	2	ICG_pre_	146	0.383	0.026	0.014	45.93	< 0.001	0.44**	0.05	0.56	0.001
		ΔWAI-S_bond_	146						0.13*	0.05	0.17	0.018

Note. ICG = Inventory of Complicated Grief absolute change score; WAI-S_total_ = WAI-S absolute change score; WAI-S_bond_ = bond subscale absolute change score; *R*^*2*^*adj.* = R^2^ adjusted including the pre-score; *B* = regression coefficient B; *SE* = standard error; *β* = Beta-coefficient; *ΔR*^*2*^ *=* amount of variance explained by the WAI-S change score. Significance with alpha correction: ***p < .001, **p < .01, *p < .05

Other models predicting outcome variables were not significant. The WAI-S change score of the subscales goal and task did not predict the change in the ICG. The WAI-S change scores did not predict changes in any scale of the BSI, which showed significant Spearman correlations before.

## Discussion

The present study analyzes the association of changes in WA in an internet-based cognitive-behavioral intervention with grief symptoms after pregnancy loss. The applied cognitive behavioral therapy program significantly reduced symptoms of prolonged grief, traumatic stress, and overall psychopathology [46, 47]. We studied the change of WA during the program and the effect of WA change on reducing grief symptoms in a sample of parents who lost a child during pregnancy.

The present study provides evidence that WA (total scale and all subscales) increased significantly during a short intervention from sessions 4 to 10. The results are in line with two other internet-based interventions that also reported an increase in WA [30, 68]. Other studies found no significant change from mid- to post-treatment [32, 69] or did not reported any change [28, 29]. When looking at similar studies in terms of methods (point of data collection and statistics) and content (type of treatment) (see 49), differences might be explained by cultural aspects. Knaevelsrud and Maercker [30] studied PTSD in a German sample. In contrast, Wagner et al. [32] studied PTSD in an Arabic sample. Our results might be more in line with the findings of Knaevelsrud and Maercker, who studied a German sample [30]. Nevertheless, this conclusion cannot be drawn based on our sample. Future studies should engage in conducting cultural comparative studies encompassing diverse cultural samples to clarify the question of intercultural differences concerning the impact of working alliance on intervention outcomes.

To the best of our knowledge, this is the first study that correlates changes in WA – as opposed to one WA score at a specific time (e.g., mid- or post-treatment) – with treatment outcome. Concerning our second hypothesis, correlational results indicate that an increase in WA was associated with reduced grief symptoms. Regression analysis showed that increased WA predicted a decrease in grief symptoms when controlling for pretreatment grief symptom severity (hypothesis 3). The increase in the total WA accounted for 3.6% of the variance in the grief-specific symptom change. Probst et al. [21] reported in their meta-analysis over 23 studies that alliance-outcome correlations accounted for 6% o treatment outcomes variability. The explained variance in our study may be smaller because of a non-clinical sample. Furthermore, Probst et al. [21] included studies with remote face-to-face contact. This might have raised the influence of the WA on treatment outcomes.

Regarding our hypothesis 4, task- and goal-specific aspects of WA should have correlated more with grief reduction than bond-specific aspects [18, 34]. This hypothesis could not be confirmed. Correlational analysis revealed that goal-, task-, and bond-specific aspects of WA were associated with grief reduction, but only the bond-specific aspect predicted grief reduction in regression analysis. Other studies found the task- and the goal-subscale more often correlated with treatment outcome than the bond-subscale [34, 68, 70]. First of all, the non-significance result regarding the goal- and task-subscale should be interpreted cautiously because the WAI-S subscales correlate highly [71]. The non-significance of both subscales might be driven by reduced statistical power. Moreover, the variance in those subscales might have been slightly lower than in the other subscales due to clear instructions before the program’s start. Due to the more standardized instructions in the internet-based therapy, there may have been less uncertainty regarding the goals. Another explanation could lie in the particular characteristics of grief-focused interventions: Given the sensitive nature of grief [35] and prenatal loss in particular, the emotional bond between therapist and participant may play a particularly crucial role. Hence, treating grief after pregnancy loss might benefit more from focusing on bond-related aspects. A meta-analysis stating that bond related aspects might be slightly less important than task-oriented aspects [21] only focused on interventions other than grief-related. There might be still limited understanding regarding the specific significance of the working alliance in interventions addressing grief related to perinatal loss, as well as its influence on bereavement interventions overall [36]. Future studies should therefore focus on examining the influence of the subscales on outcomes in disorder-specific interventions.

Regarding our hypothesis 5, WA was not associated with posttraumatic stress and its subscales, although internet-based grief interventions were found to reduce posttraumatic stress symptoms even stronger than grief symptoms [39, 40]. Similar results applied to the other symptom outcomes. According to hypothesis 6, an increase in WA correlated with a reduction in global symptom severity. Only the WA change in the task subscale was not associated with decreasing global symptom severity. The change of WA was not of predictive value for global mental health change. Similar findings were found regarding hypotheses 7 and 8. The increase in WA was correlated with a reduction in depression for the total scale as well as the subscales goal and bond. A reduction in anxiety symptoms was correlated with changes only in the goal subscale. These results should be interpreted with caution because the regression analysis did not confirm any predictive value of all WA scales. Since these results are inconclusive, future research should further differentiate the symptom-specific effects of WA in internet-based therapies for grief.

To summarize, we found an increase in the WA and all its subscales during the program. The increase in WA predicts, to some extent, a reduction of prolonged grief symptoms. Our results are in line with the effect sizes of meta-analytical data for the influence of WA on treatment outcomes [19].

*Limitations*.

Our results should be interpreted considering some limitations. First, we observed ceiling effects for WA. The ceiling effects may have reduced variance and led to type 2 errors in regression analysis. Further studies could use visual analog scales to avoid ceiling effects in measuring WA [72].

Regarding the measurement of the WA, we would like to make two critical remarks. First, the German translation of the questionnaire used was not validated. The structural validity of the measurement instrument could be questioned.

Second, the WA in our paper directly refers to the therapist of the internet-based intervention. In a few studies, instruction was changed slightly, and the goal- and task-specific items were related to the online program instead of the therapist (e.g., [71, 76]). This could have strengthened or weakened the alliance-outcome association contingent upon program and therapist contact. Further studies should use the validated and adjusted Working Alliance Inventory for guided Internet interventions (WAI-I) by Gómez Penedo et al. [74] to appropriately measure task- and goal-specific aspects of the interventions.

Third, we measured the WA only two times at mid- and post-treatment. Future Studies should consider measuring WA at multiple points to gain a deeper insight into the changes in WA throughout the course of the intervention. The frequent measuring of WA (see [25, 73–75]) would enable observing WA’s course during the program [76] and would assure linking WA to intervention techniques.

The sample predominantly consisted of female participants, which minimizes the generalizability of our results. Furthermore, the analysis has concentrated on grief symptoms, which were assessed through the Inventory of Complicated Grief (ICG). However, the ICG does not wholly cover current criteria for prolonged grief according to DSM-5 or ICD-11 [77]. Future studies may use the Traumatic Grief Inventory Self-Report Version (TGI-SR) [78].

## Conclusions

The present study contributes to the existing research in the field of alliance-outcome association and expands empirical evidence for the role of changes in WA in grief-addressing internet-based interventions. Our findings suggest that WA increases in total and all subscales upon mid-treatment and that this increase is associated with grief reduction. Open questions remain concerning the detailed course of WA, the possible influence of intervention techniques on WA, and the detailed moderators of alliance-outcome association. Further clarification of these questions might be necessary for future interventions to deepen the understanding of differential results regarding the relationship and impact of WA on treatment outcomes.

### Electronic supplementary material

Below is the link to the electronic supplementary material.

Supplementary Material 1

## Data Availability

The data supporting this study’s findings are available from the corresponding author upon reasonable request.

## Abbreviations

BSI: Brief Symptom Inventory
GSI: Global Severity Index
ICG: Inventory of Complicated Grief
IES-R: Impact of Event Scale–Revised
MCAR: Missing completely at random
PTSD: posttraumatic stress disorder
TG: treatment group
WA: working alliance
WAI-S: Working Alliance Inventory–short form
WCG: waiting control group
