# Supplementary material for: The change of working alliance and the association to treatment outcome in an internet-based therapy after pregnancy loss
Source: BMC Psychol. 2024 May 7;12:254. doi: 10.1186/s40359-024-01751-4 (PMC11077727; doi:10.1186/s40359-024-01751-4)
Supplement: Supplementary file 1 — Supplementary Material 1 [file 40359_2024_1751_MOESM1_ESM.docx]

**Appendix**

| **Table A.** Different therapists and their number of participants treated. | | |
| --- | --- | --- |
| Therapist | Number of participants treated | Percentage of participants treated |
| 1 | 20 | 13.7 |
| 2 | 37 | 25.3 |
| 3 | 9 | 6.2 |
| 4 | 22 | 15.1 |
| 5 | 38 | 26 |
| 6 | 16 | 11 |
| 7 | 3 | 2.1 |
| unknown | 1 | 0.7 |
| total | 146 | 100 |

| **Table B.** Statistical analysis of differences between completers and dropouts at baseline survey. | | | | | | | | |
| --- | --- | --- | --- | --- | --- | --- | --- | --- |
| Variable | Group | *n* | *M* | *SD* | *t^a^* | *χ^2 b^* | *df* | *p* |
| Age | Completer | 146 | 34.50 | 4.91 | 2.76** |  | 43.82 | .008 |
|  | Dropout | 32 | 31.72 | 5.21 |  |  |  |  |
| Gender (female, male) | Completer | 146 |  |  |  | 1.01 | 1 | .316 |
|  | Dropout | 32 |  |  |  |  |  |  |
| Level of education (low, middle, high) | Completer | 146 |  |  |  | 2.22 | 2 | .329 |
|  | Dropout | 32 |  |  |  |  |  |  |
| Years of education (years) | Completer | 146 | 17.67 | 2.56 | 0.24 |  | 41.44 | .809 |
|  | Dropout | 32 | 17.53 | 2.99 |  |  |  |  |
| Employment (< 15, 15 to 34, > 34 h/week) | Completer | 138 |  |  |  | 5.63 | 2 | .060 |
|  | Dropout | 30 |  |  |  |  |  |  |
| Strength of religiousness | Completer | 142 | 2.43 | 1.13 | 2.10* |  | 53.44 | .041 |
|  | Dropout | 32 | 2.03 | 0.93 |  |  |  |  |
| Time since loss (weeks) | Completer | 143 | 58.21 | 127.00 | 2.14* |  | 163.37 | .034 |
|  | Dropout | 28 | 32.40 | 30.52 |  |  |  |  |
| Time of loss (week of pregnancy) | Completer | 146 | 18.16 | 10.68 | -1.22 |  | 46.84 | .229 |
|  | Dropout | 32 | 14.97 | 6.96 |  |  |  |  |
| Number of previous miscarriages | Completer | 46 | 1.59 | 1.13 | -0.98 |  | 11.98 | .346 |
|  | Dropout | 11 | 1.00 | 0.45 |  |  |  |  |
| Treatment duration (weeks) | Completer | 135 | 6.60 | 5.14 | -0.51 |  | 29.90 | .617 |
|  | Dropout | 24 | 7.24 | 5.75 |  |  |  |  |
| Prolonged grief (ICG) | Completer | 146 | 2.53 | 0.63 | -0.68 |  | 41.61 | .501 |
|  | Dropout | 32 | 2.63 | 0.73 |  |  |  |  |
| Depressive symptoms (BSI_depression_) | Completer | 146 | 1.17 | 0.80 | -1.52 |  | 40.39 | .138 |
|  | Dropout | 30 | 1.44 | 0.91 |  |  |  |  |
| Anxiety Symptoms (BSI_anxiety_) | Completer | 146 | 0.72 | 0.67 | 0.08 |  | 48.32 | .939 |
|  | Dropout | 30 | 0.71 | 0.58 |  |  |  |  |
| General mental health (BSI_GSI_) | Completer | 146 | 0.84 | 0.51 | -1.05 |  | 40.97 | .299 |
|  | Dropout | 30 | 0.96 | 0.57 |  |  |  |  |
| Traumatic stress (IES-R) | Completer | 146 | 1.96 | 0.70 | -0.93 |  | 38.97 | .357 |
|  | Dropout | 32 | 2.12 | 0.93 |  |  |  |  |
| Working Alliance middle (WAI-S_mid_) | Completer | 146 | 2.53 | 0.63 | -0.68 |  | 41.61 | .501 |
|  | Dropout | 20 | 5.73 | 0.88 |  |  |  |  |
| Working Alliance post (WAI-S_post_) | Completer | 146 | 6.14 | 0.69 | - |  | - | - |
|  | Dropout | 1 | 6.42 | - |  |  |  |  |
| *Note. ^a^*Welch's t-test, ^b^Pearson-χ2-test with asymptotic significance. Significance (two-tailed) without alpha correction: ****p* < .001, ***p* < 0.01, **p* < .05. | | | | | | | | |

| **Table C.** Statistical analysis of differences between completers and participants with missing values at scale level at baseline survey. | | | | | | | | |
| --- | --- | --- | --- | --- | --- | --- | --- | --- |
| Variable | Group | *n* | *M* | *SD* | *t^a^* | *χ^2 b^* | *df* | *p* |
| Age | Completer | 146 | 34.53 | 4.91 | -0.18 |  | 41.74 | .861 |
|  | Missings | 32 | 34.72 | 5.69 |  |  |  |  |
| Gender | Completer | 146 |  |  |  | 2.56 | 1 | .110 |
|  | Missings | 29 |  |  |  |  |  |  |
| Level of education | Completer | 146 |  |  |  | 2.37 | 2 | .305 |
|  | Missings | 29 |  |  |  |  |  |  |
| Years of education | Completer | 146 | 17.70 | 2.54 | 1.66 |  | 40.48 | .105 |
|  | Missings | 31 | 16.77 | 2.88 |  |  |  |  |
| Employment (< 15, 15 to 34, > 34 h/week) | Completer | 138 |  |  |  | 4.72 | 2 | .095 |
|  | Dropout | 27 |  |  |  |  |  |  |
| Strength of religiousness | Completer | 141 | 2.43 | 1.13 | 0.62 |  | 44.31 | .540 |
|  | Dropout | 30 | 2.30 | 1.06 |  |  |  |  |
| Time since loss (weeks) | Completer | 142 | 58.00 | 127.43 | -0.44 |  | 46.33 | .659 |
|  | Missings | 31 | 68.61 | 118.84 |  |  |  |  |
| Time of loss (week of pregnancy) | Completer | 146 | 18.22 | 10.69 | -1.22 |  | 46.84 | .229 |
|  | Missings | 32 | 20.69 | 10.29 |  |  |  |  |
| Number of previous miscarriages | Completer | 45 | 1.56 | 1.12 | -0.98 |  | 11.98 | .346 |
|  | Missings | 10 | 2.00 | 1.33 |  |  |  |  |
| Treatment duration (weeks) | Completer | 134 | 6.57 | 5.14 | 0.20 |  | 86.32 | .841 |
|  | Missings | 31 | 6.43 | 2.73 |  |  |  |  |
| Prolonged grief (ICG) | Completer | 146 | 2.53 | 0.62 | -1.56 |  | 42.58 | .126 |
|  | Missings | 32 | 2.73 | 0.70 |  |  |  |  |
| Depressive symptoms (BSI_depression_) | Completer | 146 | 1.16 | 0.79 |  |  | 43.22 | .637 |
|  | Missings | 30 | 1.24 | 0.76 |  |  |  |  |
| Anxiety symptoms (BSI_anxiety_) | Completer | 146 | 0.72 | 0.67 | 0.30 |  | 58.69 | .763 |
|  | Missings | 30 | 0.69 | 0.45 |  |  |  |  |
| General mental health (BSI_GSI_) | Completer | 146 | 0.84 | 0.50 | 0.32 |  | 47.19 | .752 |
|  | Missings | 30 | 0.81 | 0.43 |  |  |  |  |
| Traumatic stress (IES-R) | Completer | 146 | 1.94 | 0.69 | -0.73 |  | 39.90 | .470 |
|  | Missings | 32 | 2.06 | 0.87 |  |  |  |  |
| Working alliance mid-treatment (WAI-S_total, mid_) | Completer | 146 | 5.87 | 0.80 | -1.07 |  | 36.84 | .290 |
|  | Missings | 25 | 6.03 | 0.66 |  |  |  |  |
| Working alliance post-treatment (WAI-S_total, post_) | Completer | 146 | 6.14 | 0.69 | -0.75 |  | 14.75 | .466 |
|  | Missings | 14 | 6.31 | 0.84 |  |  |  |  |
| *Note. ^a^*Welch's t-test, ^b^Pearson-χ2-test with asymptotic significance, Significance (two-tailed) without alpha correction: ****p* < .001, ***p* < 0.01, **p* < .05. | | | | | | | | |

| **Table D.** Analysis of missing values at item level. | | | | | | | | | |
| --- | --- | --- | --- | --- | --- | --- | --- | --- | --- |
|  | Total number of missings | Number of participants with missing values | | | | | Little’s MCAR^c^ test | | |
|  |  | 1 missing | 2 missings | 3 missings | 4 missings | 5 missings | *χ²* | *df* | *p* |
| ICG_pre_ | no missings at all |  |  |  |  |  |  |  |  |
| ICG_post_ | 4 | 4 |  |  |  |  | 91.97 | 80 | .170 |
| BSI_pre_ | 36 | 11 | 7 | 2 |  | 1 | 1.019.50 | 973 | .146 |
| BSI_post_ | 14 | 12 | 1 |  |  |  | 741.94** | 623 | .001 |
| IES-R_pre_ | no missings at all |  |  |  |  |  |  |  |  |
| IES-R_post_ | 2 | 2 |  |  |  |  | 53.29 | 42 | .114 |
| WAI-S_total, mid_ | 21 | 8 | 2 | 3 |  |  | 111.53 | 92 | .081 |
| WAI-S_total, post_ | 2 | 2 |  |  |  |  | 73.04*** | 22 | <.001 |
| WAI-S_goal, mid_ | 8 | 8 |  |  |  |  | 1.42 | 6 | .964 |
| WAI-S_task, mid_ | 4 | 2 | 1 |  |  |  | 20.44** | 5 | .001 |
| WAI-S_bond, mid_ | 9 | 3 | 3 |  |  |  | 11.35 | 13 | .582 |
| WAI-S_goal, post_ | 1 | 1 |  |  |  |  | 32.64*** | 3 | <.001 |
| WAI-S_task, post_ | no missings at all |  |  |  |  |  |  |  |  |
| WAI-S_bond, post_ | 1 | 1 |  |  |  |  | 5.76 | 3 | .124 |
| *Note.* ^c^Missing completely at random. Significance without alpha correction: ****p* < .001, ***p* < 0.01, **p* < .05. | | | | | | | | | |

| **Table E**. Statistical analysis of differences between treatment and waiting control group. | | | | | | | | | | |
| --- | --- | --- | --- | --- | --- | --- | --- | --- | --- | --- |
|  | | | | | | | | | Levene's Test of Equality of Variances | |
| Variable | Group | *n* | *M* | *SD* | *t^a^* | *χ^2 b^* | *df* | *p* | *F* | *p* |
| Age | Treatment | 83 | 34.05 | 4.74 | -1.36 |  | 144 | .177 | 0.73 | .395 |
|  | Waiting controls | 63 | 35.16 | 5.10 |  |  |  |  |  |  |
| Gender | Treatment | 83 |  |  |  | 0.25 | 1 | .617 |  |  |
|  | Waiting controls | 63 |  |  |  |  |  |  |  |  |
| Level of education | Treatment | 83 |  |  |  | 0.37 | 2 | .830 |  |  |
|  | Waiting controls | 63 |  |  |  |  |  |  |  |  |
| Years of education | Treatment | 83 | 17.78 | 2.66 | 0.44 |  | 144 | .660 | 0.05 | .828 |
|  | Waiting controls | 63 | 17.60 | 2.39 |  |  |  |  |  |  |
| Employment (< 15, 15 to 34, > 34 h/week) | Treatment | 78 |  |  |  | 0.24 | 2 | .888 |  |  |
|  | Waiting controls | 60 |  |  |  |  |  |  |  |  |
| Strength of religiousness | Treatment | 81 | 2.44 | 1.19 | 0.14 |  | 139 | .886 | 1.23 | .269 |
|  | Waiting controls | 60 | 2.42 | 1.05 |  |  |  |  |  |  |
| Time since loss (weeks) | Treatment | 82 | 44.66 | 86.32 | -1.34 |  | 82 | .185 | 4.56 | .035 |
|  | Waiting controls | 60 | 76.23 | 167.31 |  |  |  |  |  |  |
| Time of loss (week of pregnancy) | Treatment | 83 | 18.25 | 10.63 | 0.03 |  | 144 | .975 | 0.00 | .987 |
|  | Waiting controls | 63 | 18.19 | 10.85 |  |  |  |  |  |  |
| Number of previous miscarriages | Treatment | 24 | 1.71 | 1.23 | 0.98 |  | 43 | .333 | 1.13 | .295 |
|  | Waiting controls | 21 | 1.38 | 0.97 |  |  |  |  |  |  |
| Treatment duration (weeks) | Treatment | 81 | 6.71 | 2.29 | 0.41 |  | 132 | .685 | 3.79 | .054 |
|  | Waiting controls | 53 | 6.34 | 7.71 |  |  |  |  |  |  |
| Prolonged Grief (ICG_pre_) | Treatment | 83 | 2.49 | 0.62 | 0.59 |  | 144 | .556 | 0.35 | .554 |
|  | Waiting controls | 63 | 2.43 | 0.60 |  |  |  |  |  |  |
| Prolonged Grief (ICG_post_) | Treatment | 83 | 1.97 | 0.50 | 0.88 |  | 144 | .378 | 0.02 | .899 |
|  | Waiting controls | 63 | 1.89 | 0.50 |  |  |  |  |  |  |
| Depressive symptoms (BSI_depression, pre_) | Treatment | 83 | 1.14 | 0.84 | 1.11 |  | 144 | .267 | 1.86 | .175 |
|  | Waiting controls | 63 | 0.99 | 0.72 |  |  |  |  |  |  |
| Depressive symptoms (BSI_depression, post_) | Treatment | 83 | 0.50 | 0.53 | 0.27 |  | 144 | .784 | 0.11 | .744 |
|  | Waiting controls | 63 | 0.48 | 0.53 |  |  |  |  |  |  |
| Anxiety symptoms (BSI_anxiety, pre_) | Treatment | 83 | 0.73 | 0.68 | 0.95 |  | 144 | .342 | 0.08 | .785 |
|  | Waiting controls | 63 | 0.62 | 0.71 |  |  |  |  |  |  |
| Anxiety symptoms (BSI_anxiety, post_) | Treatment | 83 | 0.34 | 0.41 | 1.20 |  | 144 | .231 | 1.41 | .238 |
|  | Waiting controls | 63 | 0.27 | 0.35 |  |  |  |  |  |  |
| General mental health (BSI_GSI, pre_) | Treatment | 83 | 0.83 | 0.56 | 0.90 |  | 144 | .371 | 1.05 | .307 |
|  | Waiting controls | 63 | 0.75 | 0.50 |  |  |  |  |  |  |
| General mental health (BSI_GSI, post_) | Treatment | 83 | 0.41 | 0.38 | 0.57 |  | 144 | .568 | 0.71 | .399 |
|  | Waiting controls | 63 | 0.37 | 0.33 |  |  |  |  |  |  |
| Traumatic stress (IES-R_total, pre_) | Treatment | 83 | 1.92 | 0.71 | 1.21 |  | 0.1 | .228 | 1.67 | .199 |
|  | Waiting controls | 63 | 1.78 | 0.66 |  |  |  |  |  |  |
| Traumatic stress (IES-R_total, post_) | Treatment | 83 | 0.92 | 0.66 | 0.23 |  | 144 | .823 | 0.18 | .670 |
|  | Waiting controls | 63 | 0.89 | 0.62 |  |  |  |  |  |  |
| Working alliance (WAI-S_total, mid_) | Treatment | 83 | 5.83 | 0.84 | -0.77 |  | 144 | .440 | 1.06 | .305 |
|  | Waiting controls | 63 | 5.93 | 0.73 |  |  |  |  |  |  |
| Working alliance (WAI-S_total, post_) | Treatment | 83 | 6.06 | 0.76 | -1.45 |  | 144 | .149 | 3.46 | .065 |
|  | Waiting controls | 63 | 6.23 | 0.58 |  |  |  |  |  |  |
| *Note.* pre = pre-treatment, mid = mid-treatment, post = post-treatment. ^a^unpaired student t-test in case of equal variances, otherwise Welch's t-test., ^b^Pearson-χ2-test with asymptotic significance, Significance (two-tailed) without alpha correction: ****p* < .001, ***p* < 0.01, **p* < .05. | | | | | | | | | | |

| **Table F.** Prediction model of changes of outcome variables by changes in working alliance. | | | | | | | | | | | | | | | | | |
| --- | --- | --- | --- | --- | --- | --- | --- | --- | --- | --- | --- | --- | --- | --- | --- | --- | --- |
| Variable | Block | Predictor | *n* | *R^2^ adj.* | *ΔR^2^* | *p_F-Change_* | *F* | *df_1_* | *df_2_* | *p* | *AIC* | *BIC* | *B* | *SE* | *β* | *p* | *VIF* |
| ΔICG | 1 | ICG_pre_ | 146 | 0.360 | 0.365 | <0.001 | 82.66 | 1 | 144 | <.001 | -277.49 | -271.53 | 0.47** | 0.05 | 0.60 | .001 | 1.00 |
|  | 2 | ICG_pre_ | 146 | 0.392 | 0.036 | 0.004 | 47.73 | 2 | 143 | <.001 | -283.92 | -274.97 | 0.44** | 0.05 | 0.56 | .001 | 1.07 |
|  |  | ΔWAI-S_total_ | 146 |  |  |  |  |  |  |  |  |  | 0.19** | 0.07 | 0.20 | .006 | 1.07 |
| ΔICG | 1 | ICG_pre_ | 146 | 0.360 | 0.365 | <0.001 | 82.66 | 1 | 144 | <.001 | -277.49 | -271.53 | 0.47** | 0.05 | 0.60 | .001 | 1.00 |
|  | 2 | ICG_pre_ | 146 | 0.373 | 0.017 | 0.046 | 44.22 | 2 | 143 | <.001 | -279.56 | -270.61 | 0.45** | 0.05 | 0.57 | .001 | 1.05 |
|  |  | ΔWAI-S_goal_ | 146 |  |  |  |  |  |  |  |  |  | 0.10 | 0.05 | 0.14 | .058 | 1.05 |
| ΔICG | 1 | ICG_pre_ | 146 | 0.360 | 0.365 | <0.001 | 82.66 | 1 | 144 | <.001 | -277.49 | -271.53 | 0.47** | 0.05 | 0.60 | .001 | 1.00 |
|  | 2 | ICG_pre_ | 146 | 0.373 | 0.017 | 0.050 | 44.12 | 2 | 143 | <.001 | -279.43 | -270.48 | 0.46** | 0.05 | 0.59 | .001 | 1.01 |
|  |  | ΔWAI-S_task_ | 146 |  |  |  |  |  |  |  |  |  | 0.10 | 0.05 | 0.13 | .061 | 1.01 |
| ΔICG | 1 | ICG_pre_ | 146 | 0.360 | 0.365 | <0.001 | 82.66 | 1 | 144 | <.001 | -277.49 | -271.53 | 0.47** | 0.05 | 0.60 | .001 | 1.00 |
|  | 2 | ICG_pre_ | 146 | 0.383 | 0.026 | 0.014 | 45.93 | 2 | 143 | <.001 | -281.70 | -272.75 | 0.44** | 0.05 | 0.56 | .001 | 1.06 |
|  |  | ΔWAI-S_bond_ | 146 |  |  |  |  |  |  |  |  |  | 0.13* | 0.05 | 0.17 | .018 | 1.06 |
| ΔBSI_depression_ | 1 | BSI_depression_, _pre_ | 146 | 0.581 | 0.584 | <0.001 | 202.27 | 1 | 144 | < .001 | -215.16 | -209.19 | 0.71** | 0.05 | 0.76 | .001 | 1.00 |
|  | 2 | BSI_depression_, _pre_ | 146 | 0.583 | 0.005 | 0.204 | 102.39 | 2 | 143 | < .001 | -214.81 | -205.86 | 0.69** | 0.05 | 0.75 | .001 | 1.08 |
|  |  | ΔWAI-S_total_ | 146 |  |  |  |  |  |  |  |  |  | 0.11 | 0.08 | 0.07 | .221 | 1.08 |
| ΔBSI_depression_ | 1 | BSI_depression_, _pre_ | 146 | 0.581 | 0.584 | <0.001 | 202.27 | 1 | 144 | < .001 | -215.16 | -209.19 | 0.71** | 0.05 | 0.76 | .001 | 1.00 |
|  | 2 | BSI_depression_, _pre_ | 146 | 0.585 | 0.007 | 0.123 | 103.33 | 2 | 143 | < .001 | -215.60 | -206.65 | 0.69** | 0.05 | 0.74 | .001 | 1.06 |
|  |  | ΔWAI-S_goal_ | 146 |  |  |  |  |  |  |  |  |  | 0.09 | 0.06 | 0.09 | .145 | 1.06 |
| ΔBSI_depression_ | 1 | BSI_depression_, _pre_ | 146 | 0.581 | 0.584 | <0.001 | 202.27 | 1 | 144 | < .001 | -215.16 | -209.19 | 0.71** | 0.05 | 0.76 | .001 | 1.00 |
|  | 2 | BSI_depression_, _pre_ | 146 | 0.581 | 0.003 | 0.301 | 101.73 | 2 | 143 | < .001 | -214.26 | -205.31 | 0.70** | 0.05 | 0.75 | .001 | 1.06 |
|  |  | ΔWAI-S_bond_ | 146 |  |  |  |  |  |  |  |  |  | 0.07 | 0.06 | 0.06 | .301 | 1.06 |
| ΔBSI_anxiety_ | 1 | BSI_anxiety_, _pre_ | 146 | 0.691 | 0.693 | <0.001 | 325.47 | 1 | 144 | < .001 | -311.40 | -305.43 | 0.74** | 0.04 | 0.83 | .001 | 1.00 |
|  | 2 | BSI_anxiety_, _pre_ | 146 | 0.689 | 0.000 | 0.772 | 161.74 | 2 | 143 | < .001 | -309.49 | -300.54 | 0.74** | 0.04 | 0.83 | .001 | 1.04 |
|  |  | ΔWAI-S_goal_ | 146 |  |  |  |  |  |  |  |  |  | 0.01** | 0.04 | 0.01 | .001 | 1.04 |
| ΔBSI_GSI_ | 1 | BSI_GSI, pre_ | 146 | 0.552 | 0.555 | <0.001 | 179.35 | 1 | 144 | < .001 | -338.16 | -332.20 | 0.65** | 0.05 | 0.75 | .001 | 1.00 |
|  | 2 | BSI_GSI, pre_ | 146 | 0.554 | 0.005 | 0.189 | 91.01 | 2 | 143 | < .001 | -337.93 | -328.98 | 0.63** | 0.05 | 0.73 | .001 | 1.07 |
|  |  | ΔWAI-S_total_ | 146 |  |  |  |  |  |  |  |  |  | 0.07 | 0.05 | 0.08 | .215 | 1.07 |
| ΔBSI_GSI_ | 1 | BSI_GSI, pre_ | 146 | 0.552 | 0.555 | <0.001 | 179.35 | 1 | 144 | < .001 | -338.16 | -332.20 | 0.65** | 0.05 | 0.75 | .001 | 1.00 |
|  | 2 | BSI_GSI, pre_ | 146 | 0.553 | 0.004 | 0.254 | 90.53 | 2 | 143 | < .001 | -337.50 | -328.55 | 0.64** | 0.05 | 0.73 | .001 | 1.06 |
|  |  | ΔWAI-S_goal_ | 146 |  |  |  |  |  |  |  |  |  | 0.05 | 0.04 | 0.07 | .262 | 1.06 |
| ΔBSI_GSI_ | 1 | BSI_GSI, pre_ | 146 | 0.552 | 0.555 | <0.001 | 179.35 | 1 | 144 | < .001 | -338.16 | -332.20 | 0.65** | 0.05 | 0.75 | .001 | 1.00 |
|  | 2 | BSI_GSI, pre_ | 146 | 0.553 | 0.005 | 0.208 | 90.85 | 2 | 143 | < .001 | -337.79 | -328.84 | 0.63** | 0.05 | 0.73 | .001 | 1.07 |
|  |  | ΔWAI-S_bond_ | 146 |  |  |  |  |  |  |  |  |  | 0.05 | 0.04 | 0.07 | .221 | 1.07 |
| *Note. Δ*ICG = Inventory of Complicated Grief absolute change score; ΔWAI-S_total_ = WAI-S absolute change score; ΔWAI-S_bond_ = bond subscale absolute change score; ΔWAI-S_goal_ = goal subscale absolute change score; ΔWAI-S_task_ = task subscale absolute change score; *R^2^ adj.* = R^2^ adjusted including the pre-score; *B* = regression coefficient B; *SE* = standard error; *β* = Beta-coefficient; *ΔR^2^ =* amount of variance explained by the WAI-S change score. Significance with alpha correction*:* ****p* < .001, ***p* < 0.01, **p* < .05. | | | | | | | | | | | | | | | | | |

**Checking the assumptions of hierarchical regression to predict the change in grief (ΔICG) through the independent variables ICG_pre_ and ΔWAI-S_total_**

1. Linearity of the association were checked by using partial regression diagrams.


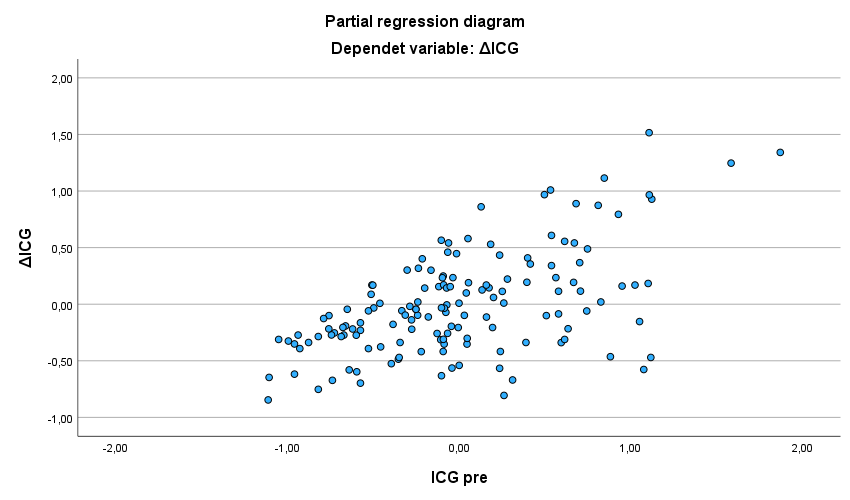

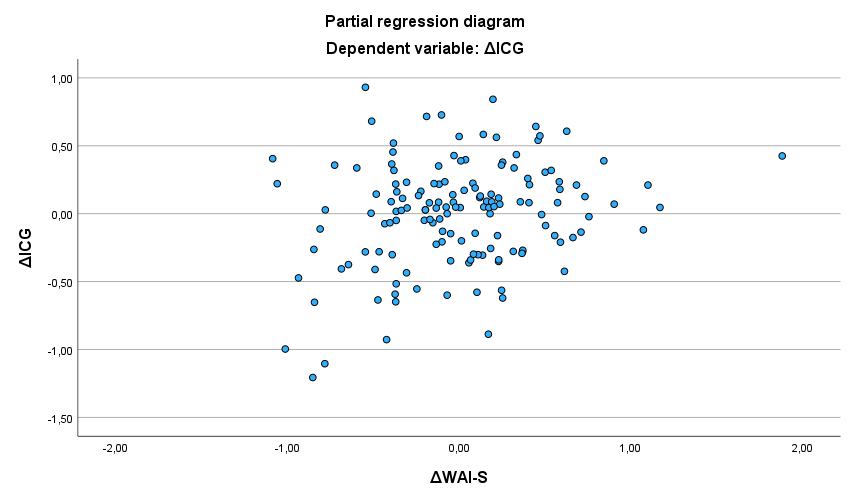


1. Outlier Analysis: Using Chebyshew’s inequality, normally or non-normally distributed data which were 3 or 4, respectively, standard deviations under or above the mean of each variable were eliminated. However, no cases were eliminated (Chernick, 2011).
2. Autocorrelation: The model had no auto-correlation as the value of the Durbin-Watson statistic was 2.096.
3. Multicollinearity: The highest value of the variance inflation factor (VIF) was 1,07 and clearly exceeded a value of 10. There was no multicollinearity.
4. Homoscedasticity of the residuals: Plotting unstandardized predicted values against the studentized residuals, the scatter plot shows almost evenly distributed values along the horizontal axis, which indicates the existence of equality of variance.


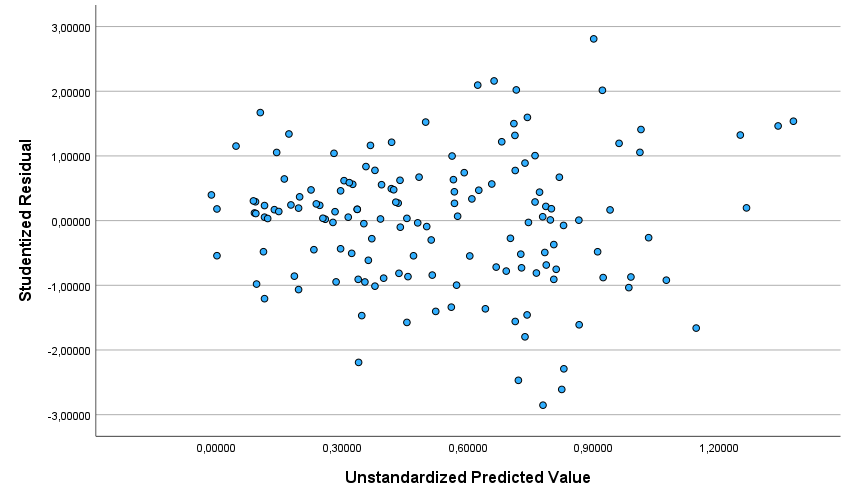


1. The normal distribution of the residuals was checked visually using the P-P-plot and the Shapiro-Wilks test. Both methods indicated a normal distribution of the residuals.

Shapiro-Wilk:

|  | Statistics | df | p |
| --- | --- | --- | --- |
| Studentized Residual | 0.990 | 146 | .408 |


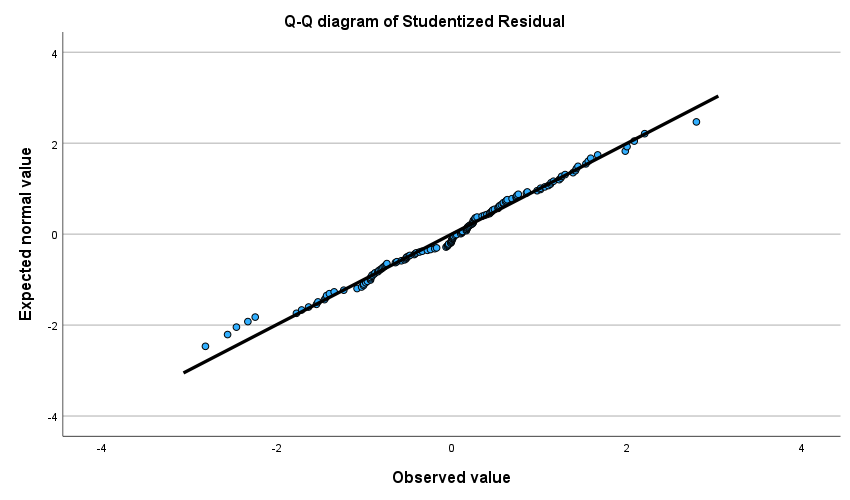


**Checking the assumptions of hierarchical regression to predict the change in grief (ΔICG) through the independent variables ICG_pre_ and ΔWAI-S_bond_**

1. Linearity of the association were checked by using partial regression diagrams.


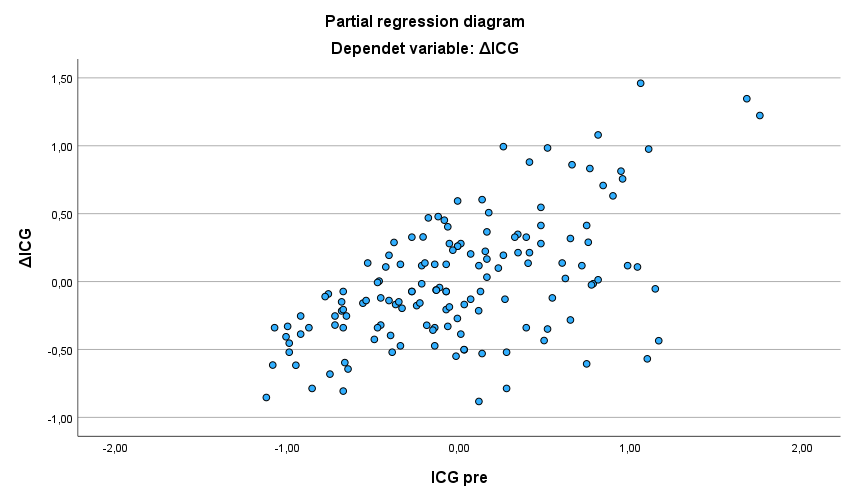

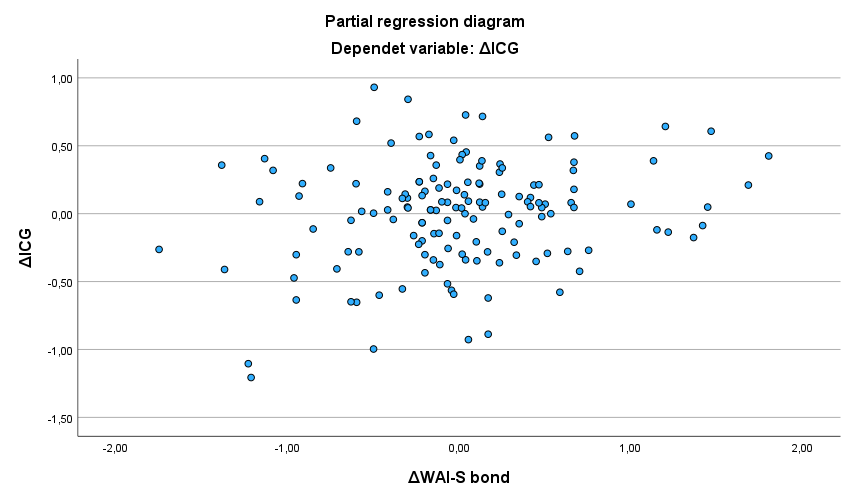


1. Outlier Analysis: Using Chebyshew’s inequality, normally or non-normally distributed data which were 3 or 4, respectively, standard deviations under or above the mean of each variable were eliminated. However, no cases were eliminated (Chernick, 2011).
2. Autocorrelation: The model had no auto-correlation as the value of the Durbin-Watson statistic was 2.067.
3. Multicollinearity: The highest value of the variance inflation factor (VIF) was 1,06 and clearly exceeded a value of 10. There was no multicollinearity.
4. Homoscedasticity of the residuals: Plotting unstandardized predicted values against the studentized residuals, the scatter plot shows almost evenly distributed values along the horizontal axis, which indicates the existence of equality of variance.


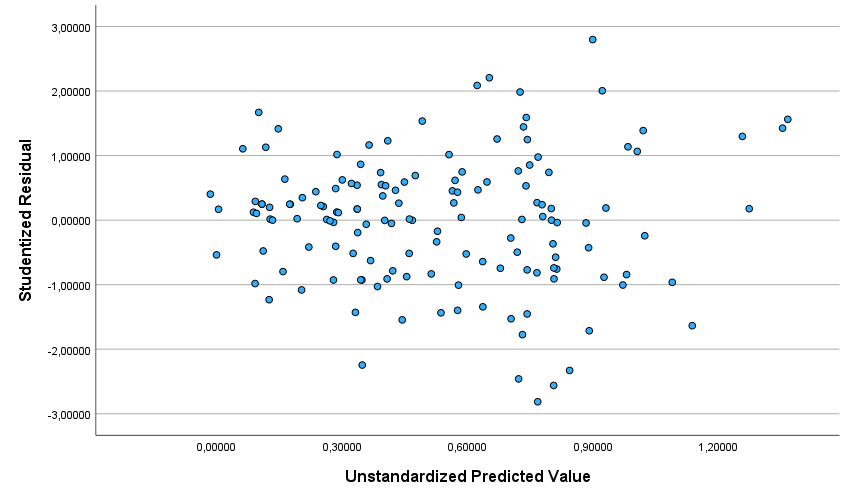


1. The normal distribution of the residuals was checked visually using the P-P-plot and the Shapiro-Wilks test. Both methods indicated a normal distribution of the residuals.

Shapiro-Wilk:

|  | Statistics | df | p |
| --- | --- | --- | --- |
| Studentized Residual | 0.989 | 146 | .341 |


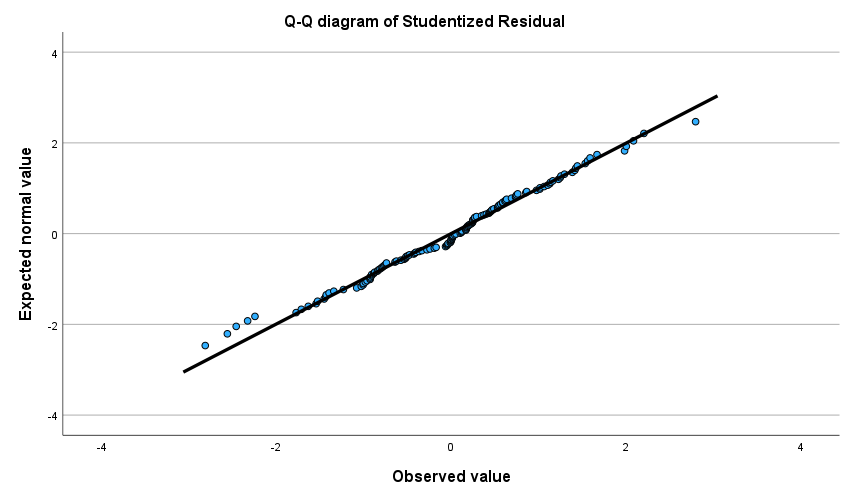


References

Chernick, M. R. (2011). *The essentials of biostatistics for physicians, nurses, and clinicians*. John Wiley & Sons.
